# Mechanisms for co-designing and co-producing health and social care: a realist synthesis

**DOI:** 10.1186/s40900-024-00638-3

**Published:** 2024-10-10

**Authors:** Daniel Masterson, Bertil Lindenfalk, Sofia Kjellström, Glenn Robert, Marlene Ockander

**Affiliations:** 1 Jönköping Academy for Improvement of Health and Welfare, School of Health and Welfare, Jönköping University, Jönköping, Sweden; 2 The School of Health Sciences, University of Skövde, Skövde, Sweden; 3 Present Address: Florence Nightingale Faculty of Nursing, Midwifery & Palliative Care, King’s College London, London, England

**Keywords:** Mechanisms, Co-design, Co-production, Participatory design, Participatory research, Healthcare, Social care, Realist synthesis, Dialogue

## Abstract

**Objectives:**

Analyse reported processes of co-design and co-production in the context of health and social care to explore the underlying mechanisms that enable inclusive and reciprocal engagement.

**Search strategy:**

Peer review research was obtained from a prior scoping review searching eight databases consisting of all methodologies relevant to co-design or co-production in the context of health and social care services and involving service-users.

**Methods of selection:**

Articles were included for synthesis if they reported a process of dialogue, with mutuality, insight and clarification in their engagement process. Ninety-three peer-review articles informed our programme theory development.

**Analysis:**

Data relating to co-design and co-production processes were extracted and analysed through inductive, abductive, and deductive analysis leading to the development of an initial programme theory.

**Main results:**

This realist synthesis finds that co-design and co-production can occur at different times, in part or all of the research and participatory process. There is an over reliance on the term ‘co-design’ or ‘co-production’ to convey complex engagement or participatory processes. We identified six mechanisms (intention, assets, dialogue, documentation, interpretation and understanding). Interaction between these six identified mechanisms in context, even if only brief, is important for supporting meaningful engagement, alignment and agreement within a co-design or co-production process.

**Implications for practice:**

The initial programme theory presented in this article provides clarity by identifying essential mechanisms which can guide the design and implementation of a range of participatory approaches. Rather than relying on a single label to convey complex participatory methods or processes, the values and principles of co-design or co-production, in combination with this programme theory, could be applied to guide implementation and reporting of specific activities within a range of research or participatory methods.

**Patient and public contribution:**

The initial programme theory was presented and piloted in a series of collaborative workshops between May 2023 and March 2024 with patient and public contributors, health professionals and researchers. This engagement process is currently underway to refine the programme theory and it is anticipated that this next phase will be completed in September 2024.

**Supplementary Information:**

The online version contains supplementary material available at 10.1186/s40900-024-00638-3.

## Abstract (summary)

The aim of this review was to understand what needs to happen for meaningful engagement to take place by exploring how co-design and co-production in health and social care has been reported in published research. Ninety-three research articles were selected as these reported engagement processes with exchanges of mutual insight. Articles which only stated that engagement took place, without reporting details, were excluded. Any information relating to co-design and co-production processes were then extracted and analysed. The findings from this review were that co-design and co-production can occur at different times, in part or all of the research and engagement process. We identified that interaction between six key elements (intention, assets, dialogue, documentation, interpretation and understanding) in context, even if only brief, are important for meaningful engagement to occur within a co-design or co-production process. We also found that there is an over reliance on the term ‘co-design’ or ‘co-production’ to convey complex processes for engagement. Rather than relying on a single word to convey such complex methods or processes, the values and principles of co-design or co-production, in combination with the key elements identified, could be applied to guide implementation, and reporting of specific activities within a range of research methods.

**Supplementary Information:**

The online version contains supplementary material available at 10.1186/s40900-024-00638-3.

## Background

During the last decade there has been an exponential growth of academic publications relating to co-design and co-production [1]. Since the introduction of the concept [2–4], co-production has come to be described as a broad umbrella term [5] covering a range of ‘co’-words [6, 7] representing various stages of co-production [8] which can be applied to a wide range of contexts, activities and actors [9]. The increasing interest in this area has led to conflation of meaning and misappropriation of “co-” words such as co-design and co-production, a phenomenon which has been referred to as a ‘co-jungle’ [10, 11] and ‘co-biquity’ [12]. Although reviews have explored the meaning of these concepts [1, 13, 14] and barriers and facilitators to co-production [14–19], few have set out to synthesise the processes and mechanisms which underpin co-design or co-production. In their rapid realist review exploring a related concept, involvement in health and care research, Ní Shé et al. [20], identified thirty-three programme theories (e.g., environmental and social planning, guidelines, and fiscal measures) that point to a range of mechanisms and resources which need to be considered. Informed by the Behaviour Change Wheel [21], these series of statements guide the development of a conducive environment for inclusion which may also inform co-production, co-design, and other participatory approaches. A realist review by Joseph-Williams et al. [22] set out to develop programme theories to explain successful use of patient decision aids in routine clinical settings. They identified eight programme theories, of which one was co-production of patient decision aids and processes with end-users. Further exploration is warranted to establish which mechanisms are present in the wider research literature in relation to co-design and co-production. Our review sets out to build upon this work by exploring the fundamental, underlying mechanisms which underpin co-design and co-production endeavours reported in the peer review literature in the context of health and social care. This realist synthesis also builds upon previous realist evaluation of two case studies, where Farr [23] provided an in-depth comparative analysis (studying the co-design of breast cancer services and co-production in local government) with a focus on power dynamics, concluding that facilitation of relational processes requires constant critical reflective practice and dialogue. During evaluation of an approach called Mental Health Experience Co-design (MH ECO), Palmer et al. [24] identified eight mechanisms of change (recognition; dialogue; cooperation; accountability; mobilisation; enactment; creativity; and attainment) as potential underlying processes which co-exist, interconnect, and interact within the context of co-design work.

### What is a realist synthesis?

Realist inquiry is a theory-driven, practice‐orientated method to examine how mechanisms and contexts interplay to produce outcomes [25]. The realist research question usually sets out to establish the underlying mechanisms (M) which produce change, the contextual factors (C) necessary to activate these mechanisms, and how the combination of context and mechanisms leads to the outcomes (O) produced [25]. According to Astbury and Leeuw [26], mechanisms are underlying entities, processes, or structures (usually hidden) which operate in specific contexts to generate outcomes of interest (p 368). This reflects the view of Dalkin et al. [27] who build upon this by noting that rather than a binary ‘on-off’ activation, mechanisms operate on a continuum. Dalkin et al. [27] define a mechanism as an exploration of how a particular intervention, programme or service in a health care setting works by changing the reasoning and resources of participants to bring about a set of intended outcomes.

### Objectives

The protocol for this realist synthesis [28] is based on the aims of the six-year research programme, ‘Samskapa’ which sets out to better understand the social processes that enable inclusive and reciprocal co-production in the context of health and social care sectors. In line with realist synthesis design [29], we aim to explore the underlying mechanisms for co-design and co-production in order to develop an initial programme theory to establish intended and unintended mechanisms for these concepts to occur in context.

## Methods

Realist inquiry was chosen as this approach allowed exploration of the interaction between context, mechanisms, and outcomes [30]. Given the complex nature of the concepts under study, this approach was most suitable for establishing what works, for whom, in what circumstances and why [31]. We report the development of a preliminary programme theory which we formulated by exploring and synthesising reported processes in the peer reviewed literature. We followed adapted PRISMA guidelines specific for realistic syntheses (RAMESES [30]). A RAMESES statement is provided in supplementary material 1. Consideration of RAMSES II [32] has also guided the continuation of the realist inquiry process.

### Searching processes

This Realist Review was primarily informed by an extensive scoping review of the existing literature which set out to establish ‘what is out there’ in the context of health and social care (Masterson et al. [1]). This realist synthesis provides in an-depth analysis of the included articles from this scoping review. The full search strategy for all included databases is provided in supplementary material 2. A full explanation of the chosen concept, key words and design of the search strategy and pilots informing these are also provided in Masterson et al. [1]. In brief, this scoping review was chosen as this explored peer reviewed research consisting of all methodologies relevant to co-design or co-production in the context of health and social care services, involving service-users and written in English. The term ‘service user’ as an inclusion criterion was chosen following consultation with patient and public contributors, defined as ‘individuals engaged in any collaborative process that extends beyond the usual, direct provider–client relationship and may consist of individuals or groups of people who identify as citizens, patients, family member or carers’ [1]. Service providers were considered to be anyone whose identified professional role and responsibilities broadly represent the delivery of health and social care services. The label of the concept, the extent of the engagement and the description of the participants who engage in co-design, co-production and related concepts varies greatly depending on the adopted definition for these concepts and the context in which they are applied [1]. Given this complexity and that we are primarily interested in reported processes of ‘co‐production’ and ‘co‐design’, we focused on these concepts in the search strategy. The search terms for the concepts were informed by seminal articles, pilot searches and a research librarian but lacked input from a patient and public contributor group. Applying an inclusive approach, search terms for the concept (co-produc* OR coproduc* OR co-design* OR codesign*) and context (health OR social OR “public service*” OR “public sector”) were broad and used in the following databases: CINAHL with Full Text (EBSCOHost), Cochrane Central Register of Controlled Trials (Wiley), MEDLINE (EBSCOhost), PsycINFO (ProQuest), PubMed (legacy), and Scopus (Elsevier). The scoping review was performed on 18th March 2019 and led to 979 included articles. In order to update the literature, a rapid review with a similar focus and strategy conducted in June/July 2022 was later incorporated. This is reported below.

#### Selection of documents

Three reviewers (DM, BL & MO) formed an independent-review triangle for this realist synthesis. All included titles and abstracts from the scoping review (*n* = 979) were distributed randomly (using Microsoft Excel) between three groups, anonymised and reviewed in Rayyan (a web- and mobile app for systematic reviews). For each Rayyan review, two reviewers read the title and abstract separately. During screening, articles were required to report the process for co-design or co-production; reviewers could assign ‘include’, ‘exclude’ or ‘maybe’. During this initial screening phase, reviewer agreement led to 227 articles being included for full text reading, 8 assigned as ‘maybe’ and put forward for full text reading and 526 being excluded.

#### Changes in the review process

**Fig. 1:**
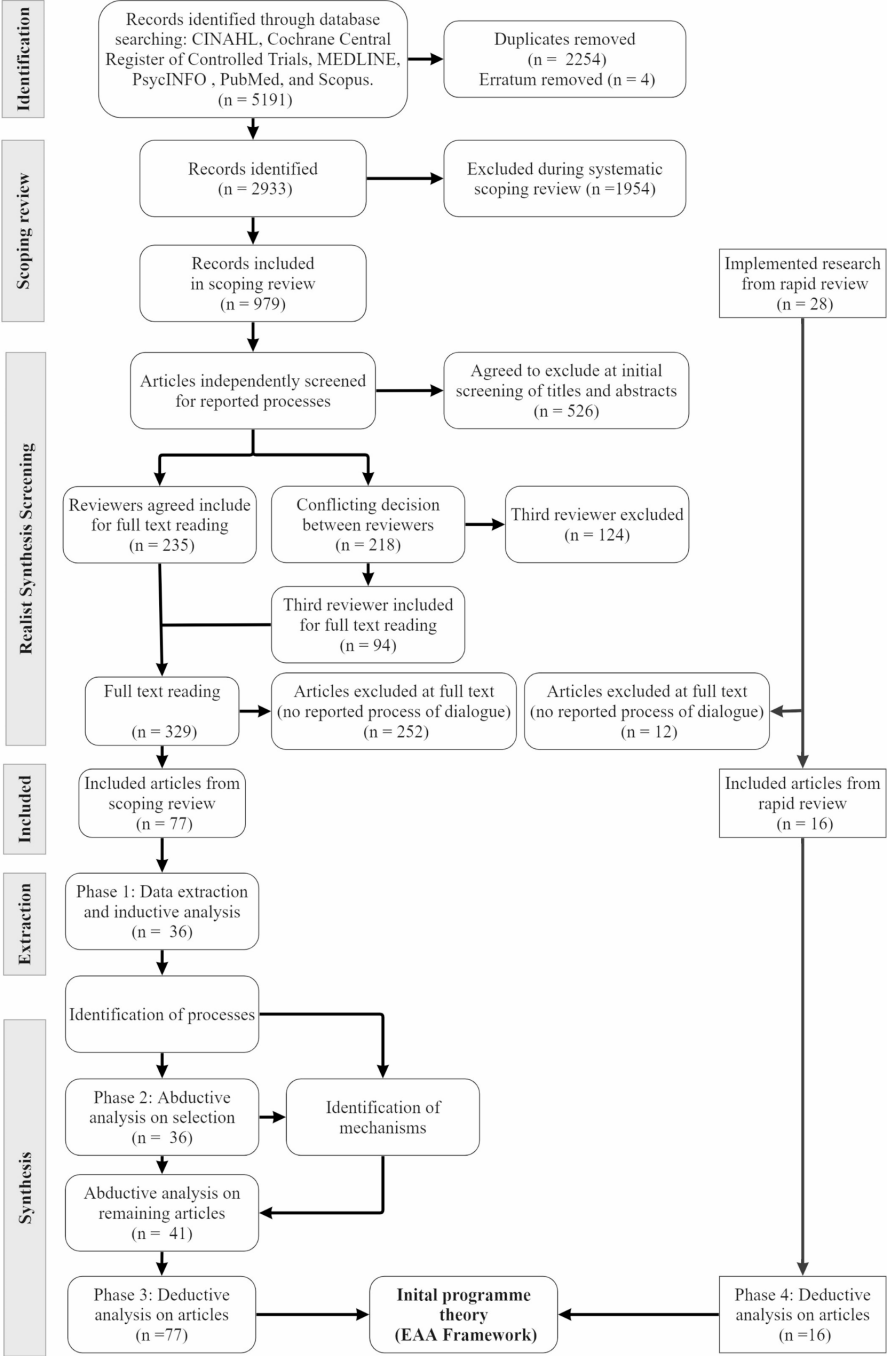
A document flow diagram

We identified 218 decision conflicts (22.3%) in the initial screening phase. Discussion of these conflicts identified the diversity in reporting processes of engagement within the peer review literature and led to a decision to adjust our inclusion criteria to require a process of dialogue to be reported. According to Senge [33], dialogue can only occur when the group have ‘a mutual quest for deeper insight and clarity’ (p. 245). Our inclusion criterion was adapted to identify articles which detailed opportunities for mutuality, insight and clarification in their engagement process. For example, stating that interviews and focus groups took place does not necessarily mean that a process of dialogue occurred. Articles were included if they went beyond a ‘one-way’ extractive process and detailed opportunities for collaborative, ‘back and forth’ interactions. The content, quantity and quality of this dialogue was not assessed. Following this change, the abstracts of the conflicts were passed onto the third reviewer for further independent review. From the 218 original decision conflicts, an additional 54 articles were then included, 40 were put forward to reading at full text as ‘maybe’ and 124 were excluded (see Fig. 1).

#### Appraisal of documents

Full text reading involved considering the updated inclusion criterion (a process of dialogue must be described) on included documents from the screening phase (*n* = 329). If an article was proposed for exclusion at full text reading, a second reviewer read to confirm this decision. In cases of conflict, a third reviewer would also review. Unlike the screening phase, which was independent, this involved interactive discussion between reviewers until consensus was achieved. Reviewers separately read and documented notes on each article which were then uploaded onto a digital whiteboard. A digital whiteboard functions like a traditional whiteboard but as an online tool it allows addition of text, digital files, drawings, and both real-time and asynchronous collaboration. Using this tool allowed reviewers to meet online, present notes on an article and debate whether the amount of information was sufficient to inform the reader about whether dialogue took place. This lengthy process took place over several meetings which spanned weeks, identifying a further 252 articles for exclusion resulting in seventy-seven included documents in the final data set from the scoping review.

### Data extraction, synthesis, and analysis

This was an iterative process consisting of four distinct phases with inductive, abductive and then deductive analysis. The included articles from the scoping review were randomly separated into two groups. Reported processes from the first group of articles (*n* = 36) were extracted and inductively analysed to inform initial identification of underlying mechanisms. The second phase involved reading the remaining articles from the second group (*n* = 41) to undertake abductive analysis to further develop the identified mechanisms and formulate a draft framework. The third phase involved conducting a deductive analysis using the developed framework to explore the reported processes in the full-text documents to identify and evidence supporting examples. A fourth phase involved repeating the deductive analysis on research articles located by a more recent rapid review.

#### Phase 1 – inductive analysis

All data reporting what had taken place and why were extracted from the first group of full articles (*n* = 36). This included any information (text or visuals) which informed preparation, implementation, processes, ways of working or any interaction relevant to co-design or co-production. The extracted data were added into a digital whiteboard and separated so that a text box represented an action or activity in the structured process. These text boxes were then arranged into rows representing each included report and rearranged into chronological order (for example, ethical considerations may have been reported at the end of the report though undertaken prior to recruitment). Through thematic analysis of the extracted processes and activities, similar activities and processes were aligned in vertical columns to form thematic categories. These columns were inductively analysed by the three reviewers to establish processes and underlying mechanisms required for co-production or co-design to occur. Authors frequently met to analyse the extracted data and then went back to the full report for broader context. Extracted data was either placed under previously identified headings or new ones were created until saturation was reached. This inductive analysis led to identification of 45 distinct processes which were grouped under seven process headings labelled: Why; Who; How; What; Refine; Result and Reflect (Table 1).

**Table 1:** Synthesis of reported processes

Why	Who	How	What	Refine	Result	Reflect
1. Spark 2. Setting 3. Objective 4. Reasoning 5. Exploration 6. Ethics	7. Strengths & resources 8. Initial recruitment 9. Secondary recruitment 10. Contributors 11. Further recruitment	12. Working definition 13. Funding 14. Materials 15. Food & drink 16. Practical assets 17. Venue 18. Power dynamics 19. Structure 20. Warm-up 21. Listening exercise 22. Develop trust 23. Recognise emotion 24. Ways of working 25. Record keeping	26. Exploring challenges 27. Knowledge gathering 28. Shared understanding 29. Idea generation 30. Knowledge giving 31. Reaching consensus 32. Prototyping	33. Validation 34. Wider recruitment 35. Feedback 36. Co-refine	37. Shared decision 38. Communication 39. Implementation 40. Launch event	41. Reflection 42. Challenges 43. Evaluation 44. Closure 45. Next steps

#### Phase 2 – abductive analysis

The synthesised data from the articles in the first data set (*n* = 36) were abductively analysed to explore underlying mechanisms by mapping the identified processes using Dalkin et al.’s [27] Context-Mechanism-Outcome (CMO) framework. Authors reviewed these extracted processes and discussed potential underlying mechanisms required for co-design or co-production to take place. This led to the identification of an adapted CMO framework with three key participatory outcomes: engagement, alignment, and agreement.

To refine the preliminary draft set of mechanisms, the reported processes within the remaining articles (*n* = 41) were then analysed using the adapted CMO framework. This analysis was an iterative process which involved discussion on insights from the previous phase, re-reading the full text to identify examples of proposed mechanisms in context, adjusting and simplifying a framework of mechanisms to reflect insights from the data. This process led to the identification of six mechanisms: intentions; assets; dialogue; documentation; interpretation; and understanding. These mechanisms are detailed further in the findings. At this stage in the analysis, these were not considered sequential steps but rather essential components which interact iteratively within a given context to achieve three outcomes (engagement, alignment, and agreement).

#### Phase 3 – deductive analysis

Once the mechanisms framework was agreed upon between the authors, deductive analysis was undertaken which involved reading the full text of all included documents. The data extraction database represented each component of the framework including underlying mechanisms and the seven processes identified (supplementary material 3). Authors went back to the original text from the extracted data to finalise labels for the underlying mechanisms and process themes.

#### Phase 4 – analysis with recent literature

To explore relevance of the findings and framework to more recent work, the included articles from a Rapid Review [34] were obtained. O’Mara-Eves et al. [34] was chosen as they aimed to map evidence and identify typologies for co-production based on values and activities, thus this had similarities with our original data source. Further, this review was chosen for pragmatic reasons as the authors had presented implemented research in their results, allowing for swifter identification of relevant articles. Their search strategy focused on SCOPUS, Web of Science and Google Scholar. Search terms were Co-production AND (Value OR Values OR Benefit). Articles were excluded if they were published prior to 2021, were protocol or conference abstracts and if they did not focus on United Kingdom settings. An additional criterion was added to exclude articles not focusing on co-production of research. It is acknowledged that the focus on co-production of research within the United Kingdom means that this rapid review is not a directly comparable to the articles located from the original, broader scoping review. However, the purpose was to test the developed model with more recent and applied peer-review research and to this end, the articles identified from their rapid review were suitable. All included articles from the rapid review which were identified as implemented research (*n* = 28) were reviewed by the first author using our exclusion criterion. Eight articles were also reviewed by the last author (MO) separately and decisions were consistent with the first author, with the exception of one article. This conflict was due to reporting quality relating to who was involved. This article was reviewed by the second author (BL) and was included in this review. Twelve articles were excluded as there was insufficient information to identify a process of dialogue. The proposed mechanisms framework was applied and occurrences within each of the sixteen articles was documented.

## Results

### Document characteristics

The included articles from the scoping review were published between 2005 and 2019 across fifteen countries. All articles from the Rapid review were undertaken in the United Kingdom and published between 2021 and June 2022. The vast majority of the research identified by the scoping review were undertaken in the United Kingdom (*n* = 36), Canada (*n* = 8), Australia (*n* = 7), and Sweden (*n* = 6). All but two articles [35, 36] were separate projects with differing contexts and participant groups reflecting the wide variety of participatory studies. Broadly, most articles were exploring changes or quality improvement to some element of a health or social care service (*n* = 32), followed by digital health (*n* = 16), research or evaluation (*n* = 12), community health (*n* = 7), assistive devices (*n* = 5) and education or training (*n* = 5). The participants involved a diverse range of groups addressing physical health and mental health, social care, or community health. All research included in this review involved patient and public contributors as this was a requirement for the original scoping review [1]. For consistency, this scoping review criteria was applied to the articles obtained from the more recent rapid review though no additional exclusions were required.

As the terms co-design and co-production were sometimes used interchangeably in the articles, concepts were assigned based on the most common term reported in each article. Broadly, the majority of included articles from the scoping review were described as co-design (*n* = 55). However, co-design and co-production were often reported as a way of working, without providing details of how this was achieved or detailing the application of any specific methods. To explore this, any reported methods and associated citations were extracted. In a similar sense that the concepts of co-design and co-production are sometimes used interchangeably, it was observed that so too were a range of participatory methods. Considering all reported methods within the scoping review and rapid review data set, thirty-eight distinct participatory procedures were identified. All reported methods were grouped by theme and summarised in Table 2. A large proportion of articles either referred to the concept (co-design or co-production) to convey their method (*n* = 24) or cited a combination of participatory methods (*n* = 15). These combined approaches were grouped based on their focus on design (*n* = 10), research (*n* = 3) or both research and design (*n* = 5) (for example, applying both participatory design and participatory action research). The majority of articles reported a *participatory* method (e.g., participatory design) which was conducted in a way which facilitated dialogue *with* people. The most common reported methods were participatory research (*n* = 14); co-production (*n* = 13); co-design (*n* = 11), community-based participatory research (*n* = 10); experience-based co-design (*n* = 9); participatory design (*n* = 8); and user-centred design (*n* = 2). Remaining methods were only reported once and are presented in Table 2. Of note, 747 of the documents from the original scoping review related to applied co-design or co-production, yet 670 of these were excluded for the purposes of this review as they did not report sufficient information to identify a process of dialogue. This suggests a reliance on the term ‘co-design’ or ‘co-production’ to convey complex engagement or participatory processes. From the Rapid Review, this is also evident with twelve of the applied research articles being excluded as they did not report a process of dialogue. This indicates a need for reporting guidelines for collaborative and participatory processes, especially in relation to these concepts. At the time of writing, the only relevant reporting guidelines available on the EQUATOR network tangentially relevant to co-design or co-production in health research are GRIPP2, which is intended for Patient and Public Involvement [37]. Of the 93 included articles, only four report to have followed the GRIPP2 reporting checklist and no other reporting guidelines relevant to participatory approaches were referenced in the data set.

**Table 2:** Number of articles for reported concept and reported methods

Concept and reported method (*n*)						Total (*n*)
Co-design articles	56		Co-production articles	37		93
Co-design with (*n* = 9) and without citation (*n* = 2)	11		Co-production with (*n* = 11) and without citation (*n* = 2)	13		24
Participatory research	3		Participatory research	11		14
Community-based research	4		Community-based research	6		10
Combined methods – design	10		-	-		10
Experience-based co-design	9		-	-		9
Participatory design	8		-	-		8
Combined methods – design & research	4		Combined methods – design & research	1		5
Combined methods – research	2		Combined methods – research	1		3
User-centred design	2		-	-		2
-	-		Assets-based community development	1		1
Co-ideation	1		-	-		1
-	-		Collective autoethnography	1		1
Human-centred design	1		-	-		1
-	-		Knowledge Café	1		1
-		-	Modified Nominal Group Technique	1		1
Patient and Public Involvement	1		-	-		1
-	-		Shared decision making	1		1

### Main findings

The results of our abductive analysis on documents (*n* = 77) which detailed a process of dialogue identified seven process themes (*why; who; how; what; refine; result; and reflect*) and six mechanisms (*intentions; assets; dialogue; documentation; interpretation; and understanding*). It is proposed that these mechanisms interact iteratively and often, repeatedly, in a given context which lead to *engagement (intentions and assets)*, *alignment* (*dialogue and documentation*) and *agreement* (*interpretation and understanding*).

The seven process themes (Why; Who; How; What; Refine; Result and Reflect) were identified through grouping 45 distinct processes reported in the first phase of analysis of documents from the scoping review (Table 1). These are not considered comprehensive or exhaustive for two reasons. The first is that the exploration of processes was undertaken to explore mechanisms for co-production which involved a proportion of articles. Secondly, due to the limited space to report processes, which is unavoidable within peer review literature, important steps may not have been reported in the documents. Nevertheless, the findings further emphasise that complex, wide-ranging collaborative processes cannot be easily distilled and represented by reference to a single concept. From the identification of need or a proposal for collaboration (a spark), there are many considerations for creating a conducive environment from reflection on resources to identification of a suitable venue and developing trust. What our preliminary findings show is that exploration of perspectives and knowledge gathering does not occur spontaneously. Equally, the processes which continue once consensus has been reached are just as complex and wide ranging, with many possible avenues of exploration which cannot be fully anticipated. Acknowledging that these initial findings are not exhaustive, the seven process headings identified from the 45 distinct processes may serve as a prompt or starting point for exploration to consider broader processes. These process themes are not considered distinct steps or sequential. It is envisaged that any collaborative process may move back and forth, have periods of blending or overlap, may not address all processes equally and may skip some altogether. Though reported processes frequently started with ‘why’ and then progressed onto ‘who’, there may be an opening ‘reflection’ which have not been reported in an article. There is likely repetition of all these processes as well as essential processes which have not been captured from the literature. The wide range of participatory approaches identified convey the complexity and the challenge in reporting such approaches and further evidence the need for reporting guidelines.

**Fig. 2:**
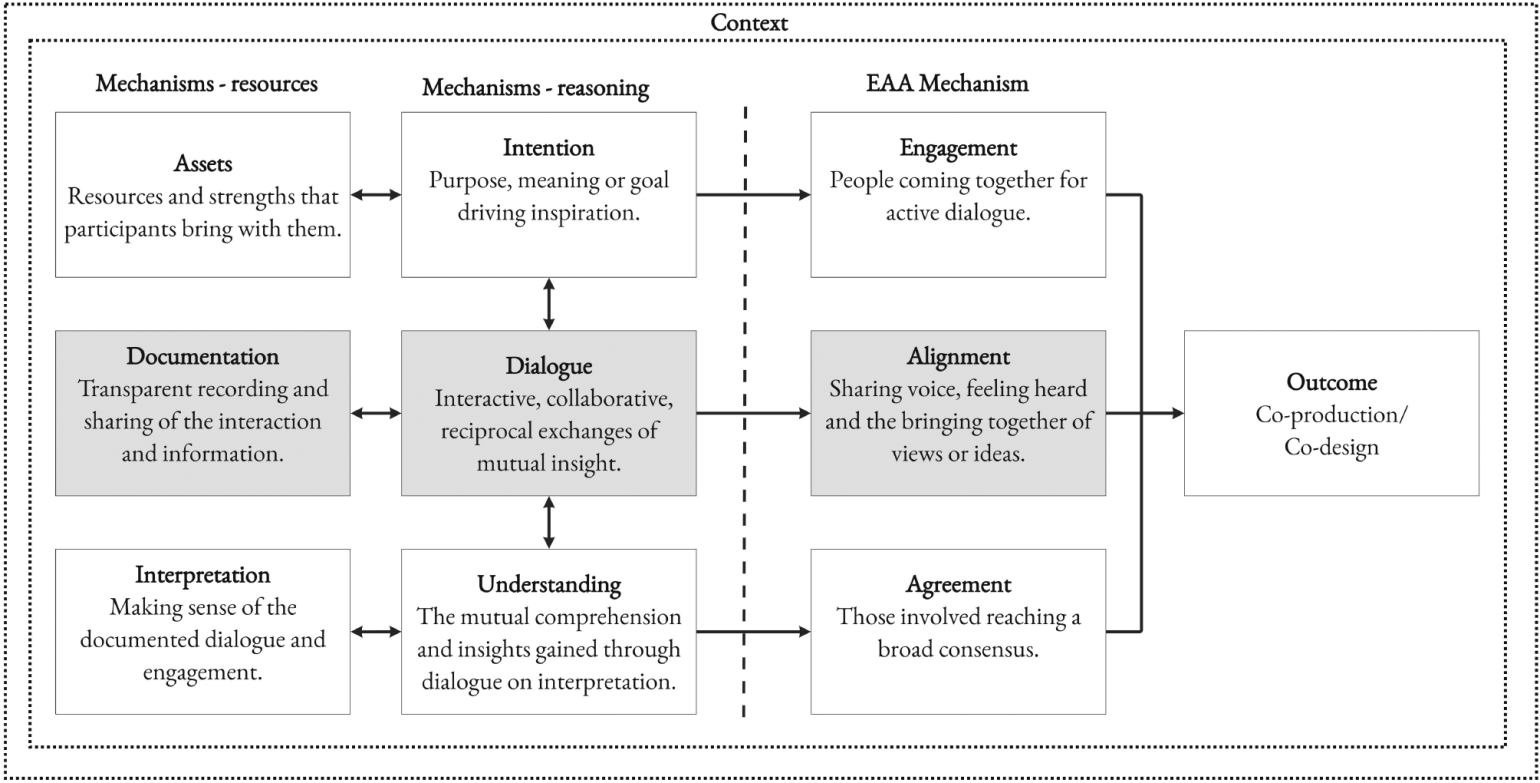
Engagement, alignment and agreement initial programme theory

**Fig. 3:**
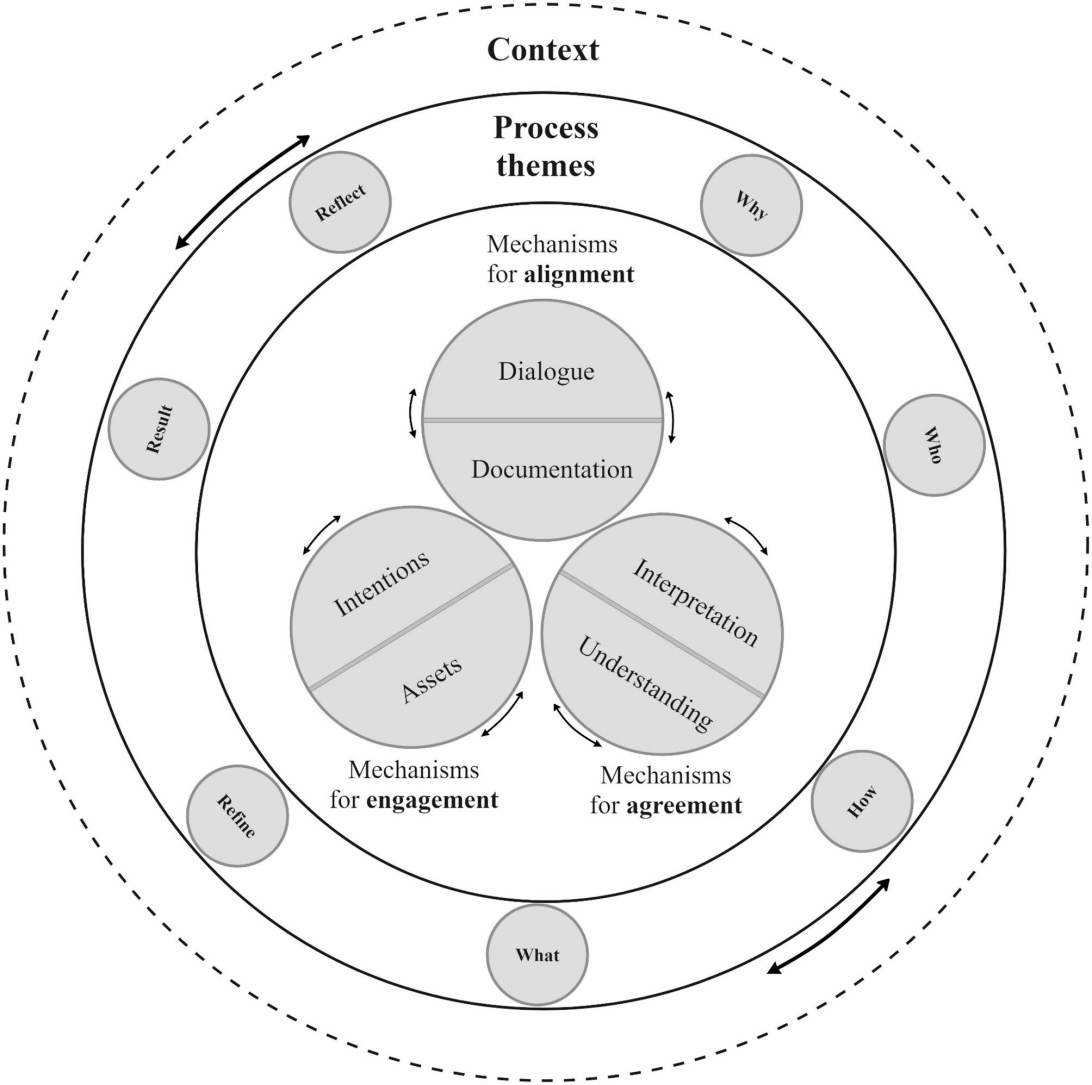
The engagement, alignment and agreement framework

The six identified mechanisms (intentions; assets; dialogue; documentation; interpretation; and understanding) interact within a given context to achieve engagement, alignment and agreement. Depending on the context, these may serve as mechanisms or each may be an outcome in of themselves. Our view is that all three are needed to interact as mechanisms in order to lead to an outcome of co-design or co-production. The initial Engagement, Alignment and Agreement programme theory, from now called the EAA framework, is presented (Fig. 2) with an accompanying accessible visual showing the interacting and iterative ‘wheels in motion’ within the identified processes (Fig. 3).

While each of the mechanisms can exist independently, it is proposed that it is the interaction between the mechanisms, even if only brief, which is important for achieving meaningful co-production in a given context. For example, initial reflection on *intentions* and *assets* (mechanisms) may create the initial conducive conditions for engagement (as an initial outcome). When there is iterative interaction with *dialogue* and *documentation* (mechanisms) this may lead to alignment on, for example, ways of working (as an initial outcome). When there are further interactions with *interpretation* and *understanding* (mechanisms) this may lead to a formal agreement (as an initial outcome) between those involved. In summary, the transformation from *intention* to *agreement* requires interaction in a given context. This interaction is not linear, rather it is an iterative and repeating interaction between all the mechanisms which overlaps and may often, repeat throughout the engagement process.

### Supporting evidence and occurrence

To exemplify the EAA framework and the complex interactions between the identified mechanisms, four examples from the included documents have been provided in supplementary material 4. These were chosen to contrast the most common methodologies reported including participatory action research; community-based participatory research; experience-based co-design; and participatory design. Table 3 presents a summary of the extracted data and provides three key insights. The first is that there is evidence of the six EAA mechanisms occurring in the reported co-production processes in the majority of the included articles. Second, these mechanisms were most often prevalent in the middle of the reported process themes (how, what and refine) with the mechanisms less evident in the earlier (why and who) and later stages of the reported process themes (result and reflect). Third, the most occurrences of the EAA mechanisms were within the *what* process theme, which refers to idea generation. The EAA mechanisms were less evident in the *why* process theme, which refers to aims and objectives. The next process theme which was lacking is in relation to *who*, which refers to identification of key individuals, persons most affected or the formalisation of a network of stakeholders. The reasons for this lack of information may be that the six mechanisms do not apply in all processes; that they do not usually take place in applied research or that they occur but are not reported transparently in the documents. It is also worth noting that the vast majority of included documents from the scoping review report processes in relation to the identified process themes *why*, *who*, *how (n = 70)* and *what (n = 77)* yet there are fewer documents reporting on *refine* (*n* = 55), *result* (*n* = 56) and *reflect* (*n* = 52). This is also reflected in the included documents from the Rapid Review. At this point, we can only speculate as to the reasons for this and future research is required to test this programme theory in practice. The synthesised data is summarised with each of the identified mechanisms in context of the seven theme headings in Table 3.

**Table 3:** Occurrence of the EAA initial programme theory within included articles

Mechanisms/Process		1. Why *n* = 70 (*n* = 16)	2. Who *n* = 70 (*n* = 16)	3. How *n* = 70 (*n* = 14)	4. What *n* = 73 (*n* = 16)	5. Refine *n* = 55 (*n* = 11)	6. Result *n* = 56 (*n* = 9)	7. Reflect *n* = 52 (*n* = 14)
**Engagement** *n* = 55 (*n* = 14)	Intentions	*n* = 28 (*n* = 14)	*n* = 21 (*n* = 8)	*n* = 29 (*n* = 6)	*n* = 41 (*n* = 7)	*n* = 22 (*n* = 3)	*n* = 15 (*n* = 2)	*n* = 7 (*n* = 5)
	Assets	*n* = 23 (*n* = 9)	*n* = 27 (*n* = 7)	*n* = 37 (*n* = 11)	*n* = 55 (*n* = 8)	*n* = 27 (*n* = 2)	*n* = 24 (*n* = 3)	*n* = 13 (*n* = 5)
**Alignment** *n* = 71 (*n* = 16)	Dialogue	*n* = 17 (*n* = 12)	*n* = 24 (*n* = 6)	*n* = 36 (*n* = 7)	*n* = 71 (*n* = 16)	*n* = 53 (*n* = 9)	*n* = 43 (*n* = 9)	*n* = 34 (*n* = 14)
	Documentation	*n* = 12 (*n* = 7)	*n* = 8 (*n* = 3)	*n* = 26 (*n* = 5)	*n* = 57 (*n* = 14)	*n* = 39 (*n* = 7)	*n* = 32 (*n* = 9)	*n* = 19 (*n* = 9)
**Agreement** *n* = 41 (*n* = 11)	Interpretation	*n* = 2 (*n* = 3)	*n* = 2 (*n* = 0)	*n* = 12 (*n* = 2)	*n* = 38 (*n* = 11)	*n* = 25 (*n* = 5)	*n* = 18 (*n* = 4)	*n* = 6 (*n* = 3)
	Understanding	*n* = 9 (*n* = 3)	*n* = 6 (*n* = 1)	*n* = 20 (*n* = 2)	*n* = 41 (*n* = 8)	*n* = 30 (*n* = 3)	*n* = 18 (*n* = 5)	*n* = 10 (*n* = 3)

n = scoping review documents, (n = rapid review documents)

## Discussion

This realist review set out to identify the reported processes for co-design and co-production to explore underlying mechanisms. We synthesised forty-five reported processes to identify six novel underlying and interacting mechanisms which co-exist and interact in context. This realist synthesis finds that co-design and co-production can occur at different times, in part or all of the research and participatory process. Further, it identifies that rather than labelling a participatory method or process as co-design or co-production, the values and principles of these concepts could be applied to guide reporting of specific activities within a range of research or participatory methods.

### Summary of main findings

Through using Dalkin’s adapted CMOc model [27], we were able to identify six mechanisms. However, it was challenging to inclusively synthesise contextual factors. Context is something which may act as a barrier, enabler, or manipulator of a mechanism [27]. It is important to note that the identified mechanisms do not interact in isolation and require complex social, physical and organisational structure to occur. While the initial EAA framework does not define context, the interaction of the six identified mechanisms within a given context determines the presence, form, duration, and application of each of these mechanisms and associated outcomes. The EAA framework has mainly been informed by collective forms of co-design or co-production but it is proposed that this framework may also take place in a single, brief interaction, such as shared decision making involving two individuals or across several, separate interactions over a period of time. The number of people involved can also differ from one-to-one interactions to several groups representing a range of perspectives. The interaction of these mechanisms may be subtle, assumed or predetermined. There may be circumstances which do not require explicit reporting but it is argued these interactions are nevertheless necessary for the mechanisms to function and for associated outcomes to be achieved. It is proposed that this EAA framework can be applied within a range of participatory methods though it is stressed that the EAA framework is not intended as methodological guidance in of itself. Rather, the process themes identified may act as a useful reminder during the planning phase or which authors can consider when setting out to report transparently. When considering the mechanisms described below it is encouraged that these are in connection with the values and principles of co-production.

#### Interaction of mechanisms in context

The result of our analysis suggests that for co-production or a co-design to occur, there needs to be some form of intention(s) and consideration of available assets. Regardless of context or concept, consideration of time and resources is required and this will likely need to be revisited, if only briefly, through a dialogic process. Any meaningful engagement also requires contemplation on intentions. The intention will differ depending on context and likely change during the engagement process. It is also likely that all mechanisms will require interaction with the dialogue and documentation mechanisms, in any given context. Dialogue occurs between two or more people and involves participation in the exploration of ideas, expression of views and active listening. During dialogue, divergence of views is required to go beyond one-way knowledge extraction. Documentation is required for alignment of dialogue to occur.

Interpretation of the documentation involves some form of reflection of the records which may take place separately or as part of the dialogic process. Interpretation can take place independently though it requires interaction with the mechanisms for alignment to move from interpretation to understanding. This understanding may be specific or broad just as it may be fleeting or more firmly established. This interaction between the six mechanisms, in a given context, is essential.

### Comparison with existing literature

Despite their distinct origins and features, the terms co-production and co-design are often used interchangeably [38] without a clear explanation of the concepts [1]. As a result of this, there have been calls for ‘meaningful’, ‘genuine’ and ‘authentic’ co-design and co-production [39] which raises the question as to what this refers. Astell and Fels [40] argue that co-production requires an environment where everyone’s voice is heard. Knowles et al. [41] argues that ‘authentic engagement’ was seen as a result of space to talk and space to change. Space to talk refers to the need to ‘create space for dialogue by explicitly recognising the importance of ongoing contributions (p. 6) and includes both agreement and disagreement. In their work exploring processes for knowledge mobilisation during patient involvement in health research, Knowles et al. [41] observe that effective co-production enables shifts in thinking and produces blended or hybrid knowledge outcomes [42, 43]. The mechanisms for engagement identified in the EAA framework links with this shift in thinking through the clarification and exploration of intentions and assets. This work also supports the findings of Farr [23] and Palmer et al. [24] who both identified the importance of dialogue in their extensive explorations of mechanisms for co-design and co-production. Recent reviews have also suggested that dialogue is an essential practice for co-production leadership [44] and leaders need to create an environment which facilitates accessible and transparent dialogue where different perspectives are valued and appreciated. This review finds that the mechanisms for alignment (dialogue and documentation) are essential for co-production to occur and that these cannot occur spontaneously. This supports the notion that these underlying mechanisms do not exist in isolation but require interconnection, which was a key finding by Palmer et al. [24] in their exploration of mechanisms for co-design. Interaction between mechanisms for alignment (dialogue and documentation) and the mechanisms for agreement (interpretation and understanding) plays an essential role in the engagement process. Palmer et al. [24] also found that their eight mechanisms co-exist within complex environment and organisational contexts, which shape the mechanisms. This review supports this finding and builds on this by suggesting additional mechanisms which facilitate dialogue. A further contribution to this area of research is identifying the importance of documenting dialogue which, though perhaps a simple consideration, has not been previously addressed within the research on this topic. Documenting divergent views offers an opportunity to reflect on what has been expressed, to review the history of decisions taken, clarify interpretation and make sure all views have been recognised. This part of the EAA framework identifies the need for interpretation and additional dialogue to have a shared understanding. Drawing from Senge [33] where an action on group level (an outcome) is a by-product of dialogue, the documentation of this dialogue is interpreted amongst group members to create an understanding of where the group is within the process, in order to decide on appropriate action. The notion of ‘shared decision-making’ is well established as being important in the co-production [45] and co-design [24] literature. Senge [33] articulates that “dialogues are diverging; they do not seek agreement, but a richer grasp of complex issues in dialogue [….] different views are presented as a means toward discovering a new view” (p247). For groups of actors representing different perspectives or various parts of the system to be able to work towards a common vision, alignment and agreement amongst actors must occur. However, this review identified that the mechanisms for agreement (interpretation and understanding) were not as frequently reported in the included articles than the mechanisms for alignment (dialogue and documentation). This gives further weight to the need for guidance on how to report, or better emphasise collaborative and participatory processes in the peer review literature. Participatory approaches such as co-production are context-dependant [46] with fixed or specific methods forming only one component of effective involvement practice [41]. As co-design and co-production do not have fixed methods or processes, but rather a practice that is flexible to fit each unique situation, there are growing calls for reporting guidelines with current work in relation to both co-creation in health and welfare [47] and co-design [48].

It is worthy of note that through this process the authors observed gaps in the model which resulted of our realist review process. These were aspects which may be important but were not supported by the literature and are therefore not evident in our initial programme theory. For example, we recognise that creativity is an important mechanism identified by Palmer et al. [24] which was not captured in this review. Knowles et al. [41] observe that while space for dialogue is partly achieved by the specific participatory methods used, it is the trusting relationship between the researcher and contributors and the openness to explore tensions which facilitates dialogue. Though likely be due to formatting and word-count constraints, there are important elements of engagement processes which are not reported in the peer-review literature. Dunston et al. [49] argues that co-production requires a dialogic and learning process with considerations of power and control. Recent work exploring the mechanisms for co-production have also identified the importance of power [23, 50] and accountability [24]. While power is incorporated in the identified process themes, it is not explicitly included in EAA framework. This is not due to a lack of importance, rather there was a lack of reported data to support inclusion. This reveals a need for guidance on reporting participatory processes in regard to power dynamics and values and principles for co-production, which are important considerations when applying the EAA framework in context. This is important to note as any method or framework can become tokenistic without such guiding principles.

Finally, evaluation was identified within the process theme of Reflection, supporting recent research which has identified the importance of measuring co-production [51]. While it is important to cite and report participatory methods, following a process alone does not guarantee meaningful co-production or co-design. This aligns with Albert et al. [46] who emphasise that participatory approaches such as co-production are context-dependant. Noting that participatory methods such as participatory design were commonly reported, this approach is also not a method or a fixed process, but rather a practice that is context-dependant, flexible to fit each unique situation [52] and to which there is no gold standard [53]. Linking with this, a further critical point to raise in regard to the initial programme theory is the challenge in accounting for contextual factors required for these mechanisms to occur. There are yet to be integrated into the model and will be a focus for continued work and is also encouraged as a consideration when applying this model in practice. It is the finding of this review that co-design and co-production are not stand-alone methods, but rather ways of working which can inform, and be informed by, a range of approaches. Given the large number of empirical articles excluded due to not reporting a dialogic process, this may indicate a need to better emphasise how collaborative and participatory processes go beyond extractive, one-way knowledge gathering or information exchange (e.g. consultancy).

In their exploration of definitions for co-design and co-production, Masterson et al. [1] recommended a shift from defining concepts in various contexts towards articulating shared values and principles underpinning both co-design and co-production. This review builds upon this by suggesting that rather than label a specific method or process as co-creation, co-design or co-production, the values and principles of these concepts could be applied and underpin a range of research or participatory methods and activities. These methods should be considered in context and reported in sufficient detail to establish how dialogue took place. This synthesis suggests that co-design and co-production can occur at different times, in part or all of the research and participatory process. Therefore, the EAA programme theory presented in this review is not intended to be followed as a stand-alone method nor intended as a quality appraisal of participatory methods. It is proposed that this EAA framework might be applied to inform a range of methods which require a dialogic process between professionals and those in need of services and guide those in creating a conducive environment for collaboration. Therefore, reflective discussions on values and principles of co-production and exploring how these inform the engagement activities and processes are encouraged. The findings of this realist synthesis will inform a realist evaluation which will both test and explore this initial programme theory with patient and public contributors and experts within this field.

### Strengths, limitations and future directions

This realist review relied on secondary data obtained from a scoping review [1] and a rapid review [34] which, though thorough, will not have captured all relevant literature and are limited to peer review research. Though this novel approach saved time in obtaining a data set, the thorough nature of the scoping review meant that data extraction and synthesis was labour intensive and time consuming because of the diversity of data in texts and the developing concept. Although this realist review has not involved an expert panel, the next steps of this realist inquiry does involve engagement with patient and public contributors, health professionals, researchers, and experts in this field to develop programmes theories using this framework as a guide. By publishing this EAA framework in the public domain in the spirit of open science principles, it is hoped application and development can help to inform other projects exploring the mechanisms for co-production and improve upon this work. The next step in the realist inquiry will be to continue to engage with patient and public contributors and experts within this field to conduct realist evaluation to explore where this programme works, with whom, in what circumstances and why.

## Conclusions and recommendations

This review has synthesised processes of co-design and co-production within the context of health and social care to identify underlying mechanisms for meaningful co-design and co-production. It was identified there is an over reliance on the term ‘co-design’ or ‘co-production’ to convey complex engagement or participatory processes. This review presents an initial programme theory which is recommended to be used to tested by those seeking engagement, alignment, and agreement when using participatory approaches in practice. This work is significant and relevant given that the majority of empirical research located in this review did not report their participatory and engagement processes. The next steps of this relist enquiry involves realist evaluation of this initial programme theory with public and patient contributors, health professionals, experts, and scholars offering critical appraisal and refinement.

## Electronic supplementary material

Below is the link to the electronic supplementary material.

**Supplementary Material 1**: RAMESES publication standards checklist.

**Supplementary Material 2**: Full search strategies for each database.

**Supplementary Material 3**: Included articles database and analyses.

**Supplementary Material 4**: Examples of four articles.

## Data Availability

The full search strategy and synthesis procedure has been provided to ensure transparency and replicability. The data that supports the main findings of this study are available in the full text of this article. The dataset containing the analysis of included articles is available as supplementary material.

## Abbreviations

CINAHL: Cumulative Index to Nursing and Allied Health Literature
EAA: Engagement, Alignment and Agreement
EQUATOR: Enhancing the Quality and Transparency of health research Network
GRIPP2: Guidance for Reporting Involvement of Patients and the Public – second version
MEDLINE: Medical Literature Analysis and Retrieval System Online
PPI: Patient and Public Involvement
RAMESES: Realist And Meta-narrative Evidence Syntheses: Evolving Standards
