# Supplementary material for: Mechanisms for co-designing and co-producing health and social care: a realist synthesis
Source: Res Involv Engagem. 2024 Oct 10;10:103. doi: 10.1186/s40900-024-00638-3 (PMC11468303; doi:10.1186/s40900-024-00638-3)
Supplement: Supplementary file 2 — Supplementary Material 2: Full search strategies for each database. [file 40900_2024_638_MOESM2_ESM.docx]

Supplementary file 2. Search strategy for each database from Masterson et al. (2022).

| **Search terms to be included in title, abstract or key words** |
| --- |
| (Co-produc* OR coproduc* OR co-design* OR codesign*)  **AND** (health OR social OR "Public Service*" OR “public sector”)  **AND NOT** (engineer* OR biomass OR bioproduct* OR osmosis OR microalgae OR ecosystems OR hydrogen OR Brand OR Algebra) |

**CINAHL with Full Text (EBSCOHost) – 588 hits on March 18, 2019**

TI ( ( co-produc* OR coproduc* OR co-design* OR codesign* ) AND ( health OR social OR “public service*” OR “public sector*” ) NOT ( engineer* OR biomass OR chemic* OR bioproduct* OR osmosis OR microalgae OR ecosystems OR hydrogen OR brand OR algebra ) ) OR AB ( ( co-produc* OR coproduc* OR co-design* OR codesign* ) AND ( health OR social OR “public service*” OR “public sector*” ) NOT ( engineer* OR biomass OR chemic* OR bioproduct* OR osmosis OR microalgae OR ecosystems OR hydrogen OR brand OR algebra ) )

**Limiters** - Peer Reviewed; English Language

**Cochrane Central Register of Controlled Trials (Wiley) – 47 hits on March 19, 2019**

(co-produc* OR coproduc* OR co-design* OR codesign*):ti,ab,kw AND (health OR social OR “public service*” OR “public sector*”):ti,ab,kw NOT (engineer* OR biomass OR chemic* OR bioproduct* OR osmosis OR microalgae OR ecosystems OR hydrogen OR brand OR algebra):ti,ab,kw

**MEDLINE (EBSCOHost) – 793 hits on March 18, 2019**

TI ( ( co-produc* OR coproduc* OR co-design* OR codesign* ) AND ( health OR social OR “public service*” OR “public sector*” ) NOT ( engineer* OR biomass OR chemic* OR bioproduct* OR osmosis OR microalgae OR ecosystems OR hydrogen OR brand OR algebra ) ) OR AB ( ( co-produc* OR coproduc* OR co-design* OR codesign* ) AND ( health OR social OR “public service*” OR “public sector*” ) NOT ( engineer* OR biomass OR chemic* OR bioproduct* OR osmosis OR microalgae OR ecosystems OR hydrogen OR brand OR algebra ) )

**Limiters** – English Language

**PsycINFO (ProQuest) – 403 hits on March 18, 2019**

ti((co-produc* OR coproduc* OR co-design* OR codesign*) AND (health OR social OR "public service*" OR "public sector*") NOT (engineer* OR biomass OR chemic* OR bioproduct* OR osmosis OR microalgae OR ecosystems OR hydrogen OR brand OR algebra)) OR ab((co-produc* OR coproduc* OR co-design* OR codesign*) AND (health OR social OR "public service*" OR "public sector*") NOT (engineer* OR biomass OR chemic* OR bioproduct* OR osmosis OR microalgae OR ecosystems OR hydrogen OR brand OR algebra))

**Limits** – Peer reviewed; Language: English

**PubMed (legacy) – 898 hits on March 18, 2019**

Search (((co-produc*[Title/Abstract] OR coproduc*[Title/Abstract] OR co-design*[Title/Abstract] OR codesign*[Title/Abstract])) AND (health[Title/Abstract] OR social[Title/Abstract] OR “public service*”[Title/Abstract] OR “public sector*”[Title/Abstract])) NOT (engineer*[Title/Abstract] OR biomass[Title/Abstract] OR chemic*[Title/Abstract] OR bioproduct*[Title/Abstract] OR osmosis[Title/Abstract] OR microalgae[Title/Abstract] OR ecosystems[Title/Abstract] OR hydrogen[Title/Abstract] OR brand[Title/Abstract] OR algebra[Title/Abstract])

**Filters:** English

**Scopus (Elsevier) – 2462 hits on March 18, 2019**

( TITLE-ABS ( co-produc* OR coproduc* OR co-design* OR codesign* ) AND TITLE-ABS ( health OR social OR "public service*" OR "public sector*" ) AND NOT TITLE-ABS ( engineer* OR biomass OR chemic* OR bioproduct* OR osmosis OR microalgae OR ecosystems OR hydrogen OR brand OR algebra ) ) AND ( LIMIT-TO ( LANGUAGE , "English" ) )
