# Supplementary material for: Mechanisms for co-designing and co-producing health and social care: a realist synthesis
Source: Res Involv Engagem. 2024 Oct 10;10:103. doi: 10.1186/s40900-024-00638-3 (PMC11468303; doi:10.1186/s40900-024-00638-3)
Supplement: Supplementary file 4 — Supplementary Material 4: Examples of four articles. [file 40900_2024_638_MOESM4_ESM.docx]

Supplementary file 4: Examples of articles analysed using EAA framework.

To exemplify the EAA framework and the complex interactions between the identified mechanisms in context, four examples from the included documents have been provided. These were chosen to contrast most common methodologies reported including participatory design, participatory action research, experience-based co-design, user-centred design, and community-based participatory research. Below is reference to the context (C), the mechanisms (M), and the outcomes (O) reported within the documents. These examples demonstrate the iterative nature of the EAA framework when applied in context. Though not intended as CMO statements, these serve an example of how the next phase of the realist inquiry will proceed in developing such statements.

**Participatory action research:** Kidd, Susan, Amanda Kenny, and Carol McKinstry. 2015. "Exploring the meaning of recovery‐oriented care: An action‐research study." International Journal of Mental Health Nursing 24 (1): 38-48.

Kidd et al. (2015) considered participatory action research useful in addressing power differences and recognising diverse perspectives (C) and used cooperative inquiry as ‘a framework to develop dialogue and build a participatory space’ (p.40). Their intention of developing dialogue (M: intention) was to draw on participants knowledge (M: assets) of recovery-oriented mental health care to encourage ‘transformation of understanding’ (p. 40). Over the course of ten, two-hour group meetings (M: assets), discussions (M: dialogue) were audiotaped and transcribed (M: documentation) which were reviewed (M: interpretation) and agreed upon (O: agreement). Thematic analysis was reported to be iterative involved the whole group (C) which involved discussing (M: dialogue), collating (M: documentation) and agreeing on final themes (O: agreement). It is interesting to note that in their main findings, the opportunity for dialogue was ‘experienced as unusual, but important’ by the participants (p.41).

**Community-based participatory research:** Vaughn, Lisa M, Farrah Jacquez, and Gabriela Suarez-Cano. 2019. "Developing and implementing a stress and coping intervention in partnership with Latino immigrant coresearchers." *Translational Issues in Psychological Science* 5 (1): 62.

Vaughn et al. (2019) worked with co-researchers as part of a community-based participatory research approach to develop a stress and coping intervention (C). A series of training sessions (M: assets) were held on scientific rigour and ethics, communication skills such as on paraphrasing, reflection on feelings, asking questions and building rapport (M: intentions). Co-researchers were involved in shared decision making (M: intention) at each phase of their research process (M: dialogue). At the end of the training sessions, co-researchers were involved in making the final decision (M: understanding) about project materials and the intervention process. Co-researchers developed and implemented the intervention (C) which involved discussion of evidence based-strategies (M: dialogue), cultural adaptations and made shared-decisions (M: understanding) on sampling, measurement, and monitoring (O: agreement). The content of the intervention was decided during five workshops (C). Agreement was reached (O: agreement) on the importance of active listening and empathy (M: assets); using ‘unlock strategies’ to identify activities which reduce stress; and goal setting during their stress reduction strategy. The authors detail a range of shared decisions (O: agreement) such as the name, logo, and identification (e.g., badges), the identifying label ‘co-researcher’s, meeting schedules, logistical support such as sustenance and training (M: assets) and ways of working. Co-researchers then delivered their intervention over the course of three one-hour sessions, maintained field notes (M: documentation) as well as the evaluation, analysis (M: dialogue), interpretation (M: interpretation) and dissemination of results.

**Experience-based co-design:** Bowen, Simon, Helena Sustar, Daniel Wolstenholme, and Andy Dearden. 2013. "Engaging teenagers productively in service design." *International journal of child-computer interaction* 1 (3-4): 71-81.

Bowen et al. (2013) explored life experiences to prompt innovation (M: intention) and motivate participants involvement (O: engagement) using Experience-based co-design (C). Prior to their project, the authors spoke (M: dialogue) to a small number of young people with diabetes and health professionals (M: assets) working with this group which established a goal to lower the threshold of engagement to services to encourage more frequent use (M: intention). When devising activities (C), Bowen et al. (2013) drew on casual conversations (M: dialogue) between the participants (M: assets). The design process involved sharing experiences (M: dialogue), exploring ‘blue sky ideas’ (O: alignment), developing practical proposals (M: documentation), prototyping and evaluating services. Prior to each workshop, an opportunity for refreshments (M: assets) and socialising (M: dialogue) was provided with the aims to build trust and rapport (M: intention) between the group (O: engagement). This project detailed eight workshops where they use a range of popular cultural references (M: assets) to promote interaction (O. engagement). In their first workshop, the authors set out to gain insight into preferences for diabetes-related equipment (M: intention) in contrast with consumer products (O. understanding). Researchers ran a ‘cool wall’ activity inspired from BBC’s Top Gear (M: assets) to prompt explanation of choices (M: dialogue) and rating (M: documentation). Their second workshop explored experiences (M: intention) using a ‘body mapping’ activity (M: assets) with three groups placing post-it notes on a silhouette (M: documentation) to explore concerns, feelings, and experiences (M: dialogue). The groups then reflected (M: interpretation) on the other group’s discussion (M: documentation). In their final workshop, they brought together the learning from previous workshops (M: understanding) to evaluate current paediatric diabetes services (C). Here they used a machinery analogy in the style popular animation series, Wallace and Gromit (M: assets) to represent the flow of information within the system over time (M: understanding). The workshop involved rating the ‘information machines’ (M: dialogue) using gauges (M: documentation) and re-design the ‘factory’ (O: Agreement) by re-arranging the machines (O: alignment). The final two workshops were held after consolidating the information (M: interpretation) from the previous workshops and to test new service designs (C) through ‘show and tell’ (M: assets) discussions (M: dialogue) before launching a welcome event trial to introduce young people recently diagnosed with diabetes.

**Participatory design:** Mirkovic, Jelena, Stian Jessen, Olöf Birna Kristjansdottir, Tonje Krogseth, Absera Teshome Koricho, and Cornelia M Ruland. 2018. "Developing technology to mobilize personal strengths in people with chronic illness: positive codesign approach." *JMIR Formative Research* 2 (1): e10774.

Mirkovic et al. (2018) explored how technology can help people manage their long-term conditions (C) reported that how they promoted meaningful roles for stakeholders in the design process (O: engagement). In their first participatory design workshop, they held an exercise to engage the participants in a shared emotional experience (M: dialogue) to help them think about personal strengths (M: assets) to draw upon during later activities (M: documentation). Mirkovic et al. (2018) reported that when stakeholders discussed ways of working (M: dialogue), rules were formulated (M: documentation) and agreed (M: understanding) to promote joint work and collaboration between participants (O: agreement). These included listening actively to others; building on each other's ideas, showing curiosity about others in the group and their ideas (Mirkovic et al., 2018, 4). In the context of idea generation (C), challenges and problems in relation to participants well-being (M: dialogue) were noted on sticky-notes (M: documentation) and presented to the wider group for voting (O: alignment). The group then ‘brainstormed’ to create new ideas (M: dialogue) and in their final session participants voted (M: documentation) on the best idea (O: agreement).

**Summary:**
These examples demonstrate that the six mechanisms within the EAA framework are reliant on interaction with each other in context, which can differ greatly. When considering how such processes are reported, our analysis indicates that dialogue and documentation appear to occur spontaneously. However, in order to achieve alignment as an outcome, there is a need to reflect on intention (e.g., a mind set for co-production) or consideration of each other’s assets (e.g., strengths, skills, resources). Further, it was observed that dialogue and documentation may occur without the exploration of the mechanisms for agreement. Without agreement on interpretation of the mechanisms for alignment, the documentation may not be an accurate representation of the dialogue or may have been misinterpreted. Without interaction with the mechanisms for engagement and the mechanisms for agreement, there is a risk that the intended dialogic process may be limited to one-way knowledge gathering.
